# The effect of an interactive mobile health intervention to improve community-based essential neonatal care practices among postpartum women in northeast Ethiopia: a cluster randomized controlled trial

**DOI:** 10.1093/inthealth/ihae080

**Published:** 2025-01-10

**Authors:** Niguss Cherie, Muluemebet Abera Wordofa, Gurmesa Tura Debelew

**Affiliations:** Reproductive and Family Health Department, School of Public Health, College of Medicine and Health Sciences, Wollo University, Dessie, Ethiopia; Population and Family Health Department, Faculty of Public Health, Institute of Health, Jimma University, Jimma, Ethiopia; Population and Family Health Department, Faculty of Public Health, Institute of Health, Jimma University, Jimma, Ethiopia; Population and Family Health Department, Faculty of Public Health, Institute of Health, Jimma University, Jimma, Ethiopia

**Keywords:** effect, mobile health, neonatal care practice, northeast Ethiopia, randomized controlled trial

## Abstract

**Background:**

Despite global declines in child mortality rates, Africa's reduction is lagging behind other regions. Neonatal survival remains a key priority in the sustainable development agenda. Promoting neonatal care practices at the individual and community levels is essential, and technology-based interventions can effectively reach potential future mothers. This study aimed to evaluate the effect of an interactive mobile health intervention on improving community-based essential neonatal care practices among postpartum women in northeast Ethiopia.

**Methods:**

This study was conducted in Dessie and Kombolcha city zones, in northeast Ethiopia. A cluster randomized controlled trial was implemented among 743 participants (376 intervention and 367 control) from 2 January to 15 June 2023. Pregnant women at 30-weeks’ gestation in selected clusters were enrolled and followed up to 45 days after childbirth. Data were collected using Open Data Kit and analysed with Stata version 17. Structural equation modelling through confirmatory factor analysis was employed. Model fitness was evaluated using the χ^2^:degree of freedom ratio, root mean square error of approximation and standardized root mean square residual, indicating a good model fit. Statistical significance was declared at a level <0.05 with a 95% confidence interval.

**Results:**

The study revealed high narrow birth-to-pregnancy intervals of <24 months in both groups (48.5% control, 49.5% intervention). Awareness of neonatal care increased markedly in the intervention group, increasing from 62.0% to 85.9%, compared with an increase from 57.8% to 67.6% in the control group. Disagreement regarding immediate newborn bathing was more prevalent in the intervention group (73.9%) than in the control group (58.9%). Initiating breastfeeding within 1 h after birth was higher in the intervention group (85.4%) compared with the control group (74.4%). Postnatal visits to health facilities were more frequent in the intervention group (79.6%) than in the control group (54.8%). Mobile health intervention (β=0.393, p=0.007) and knowledge of neonatal care (β=0.347, p=0.012) had a significant positive effect on neonatal care practices. There were no significant indirect pathways between the variables analysed. Mobile health intervention and knowledge of neonatal care remain significant predictors with a total effect of β=0.382, p=0.009 and β=0.347, p=0.012, respectively, in enhancing neonatal care practices.

**Conclusions:**

This study underscores the significant role of mobile health interventions and maternal knowledge in enhancing neonatal care practices. These findings should inform the design and implementation of maternal and child health programs, emphasizing the integration of technology and education to improve neonatal outcomes in resource-limited settings.

**Trial registration:**

Protocol Registration and Results System Clinical Trial Registry, www.ClinicalTrials.gov, NCT05666050. Registered on 23 December 2022.

## Introduction

The first 28 d is the most crucial time for a child's growth and survival.^1^ Neonatal care is vital for the health of newborns and includes maintaining body temperature through thermal care, supporting breastfeeding, ensuring proper cord care, administering immunizations and implementing strict hygiene practices to reduce infection risk.^2^ But globally, one million neonates die annually, and 99% of those deaths occur in low-income countries.^3^ Although child mortality rates are decreasing globally, the African continent is experiencing slower declines than other regions.^4^ Most of those deaths can be prevented by promotion of and providing an acceptable package of neonatal care practices.^5,6^

One major target of the Sustainable Development Goals (SDGs) is to end preventable deaths of newborns, with all countries working to decrease the neonatal mortality rate to a minimum of 12 per 1000 live births by 2030.^7–9^ The global health actors, together with researchers, policymakers and program implementers have been providing new data and technologies for neonatal survival for several years.^10^ However, the difficulty of neonatal survival remains an unfinished agenda and is among the unachieved SDG targets.^11^

Community-based interventions are necessary for reducing neonatal deaths, even where levels of facility deliveries are high.^12^ Once delivered, newborns should receive immediate newborn care, which includes thermal care (drying and wrapping, skin-to-skin contact, delayed bathing), sanitary cord care, colostrum feeding and early initiation of breastfeeding.^13^ These practices need to be promoted at the individual and community levels through various methods together with technology-based interventions to reach future potential mothers.^12^

To reduce maternal and child death, the Ethiopian government introduced several health interventions, including coaching midwives, enhancing referral systems, group action health services and implementing packages of the Health Extension Program.^13,14^ However, death rates remain high.^15^ Based on the Ethiopian Demographic and Health Survey (EDHS) reports, neonatal death has not decreased, indicating the need for new approaches to enhance neonatal care interventions and follow-up at the community level.^16–18^ To enhance the survival of neonates, community-based essential newborn care may be a priority intervention technique.^19,20^

The growth and access to mobile phones and services and the increase in mobile penetration in developing countries is anticipated to facilitate the employment of mobile health (mHealth) initiatives in resource-restricted settings.^21,22^ Extending the reach of the healthcare system, mHealth is intended to support healthcare behaviour.^23^ Major effects of a mobile health program include boosting communications between pregnant women and caregivers at different levels, recording their health status and providing necessary information to providers and users.^24–26^

There is growing evidence showing that mobile health solutions such as text messaging (Short Message Service [SMS]) improve health service delivery processes and health outcomes, notably within the areas of treatment adherence, appointment compliance and patient surveillance within the developed world.^27^ However, no evidence demonstrates the effectiveness of mHealth interventions on key maternal and child health service outcomes or neonatal care practices in Ethiopia. Thus the target of this study was to determine the effectiveness of interactive mHealth intervention, specifically the use of SMS to enhance and improve community-based essential neonatal care among postpartum mothers in the Dessie and Kombolcha zones in northeast Ethiopia. The findings of this study are expected to contribute to the existing knowledge and determine whether phone-based support for women throughout pregnancy and early postpartum improves neonatal care practices. Additionally, the findings can be used by policymakers, program implementers, non-governmental organizations, local health planners and healthcare providers as baseline information for improving healthcare services.

## Methods

### Study area, design and period

The research took place in the city zones of Dessie and Kombolcha in the Amhara Regional State in northeast Ethiopia. Dessie serves as the administrative centre of the South Wollo Zone and metropolitan city zone, situated 401 km north of Addis Ababa. Dessie is divided into five subcities with 22 kebeles and is has two governmental hospitals and eight public health centres. Kombolcha town, an industrial zone and dry port in northeast Ethiopia, is located 30 km from Dessie and 375 km from Addis Ababa. The town is organized into five subcities with 19 kebeles and possesses one governmental hospital and five health centres.^31^ For the study, a total of 20 clusters (kebeles) were randomly selected and included 10 intervention and 10 control clusters. The study employed a cluster randomized control design and was conducted from 15 January to 15 June 2023.

### Population and eligibility criteria

**Figure 1.**
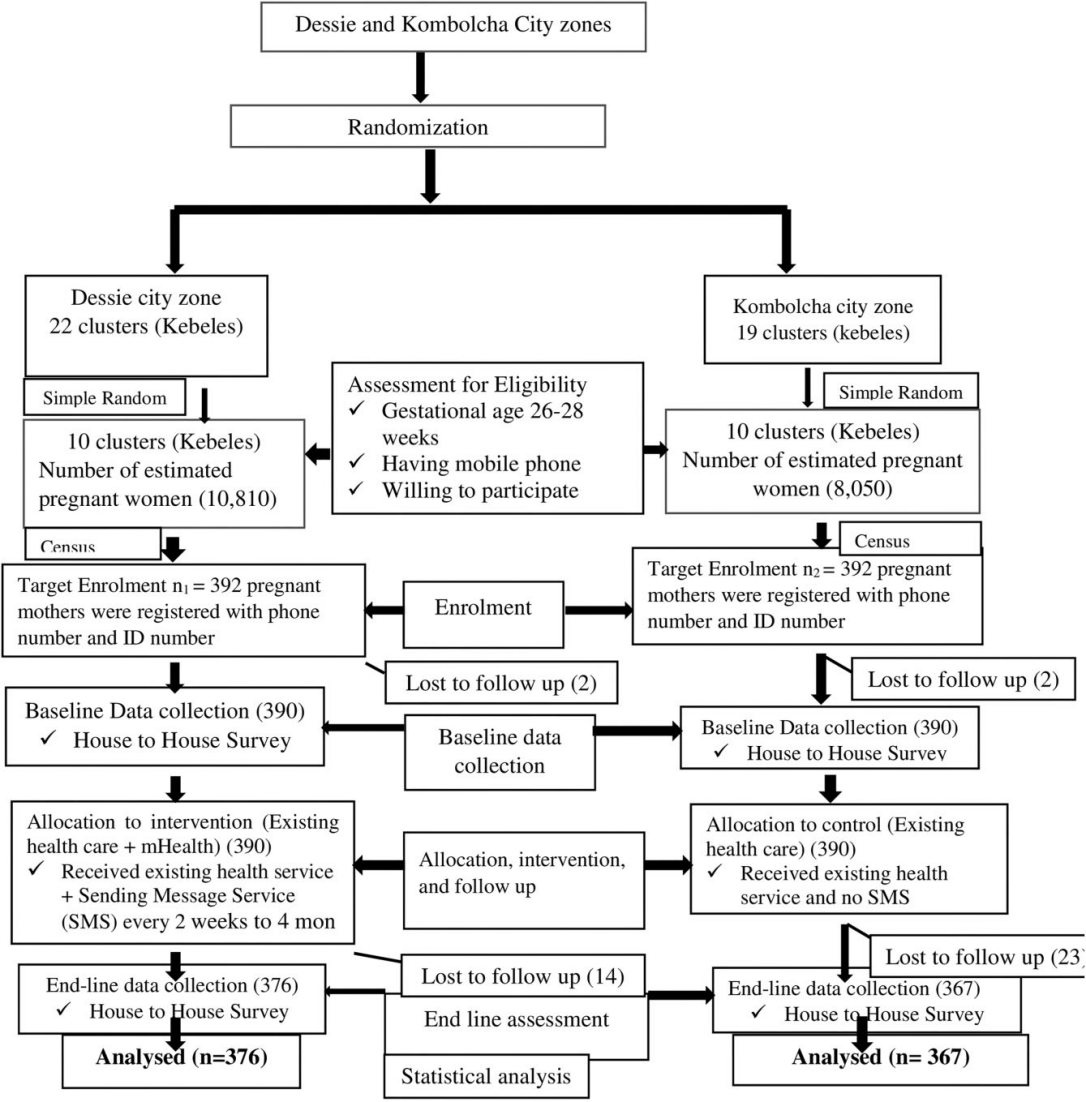
Consolidated Standards of Reporting Trials diagram depicts the sampling method, allocation of study units and assessment of eligibility criteria.

The source population for the study included all postpartum women in the designated area. During enrolment, eligibility criteria were applied and pregnant women who met the World Health Organization (WHO) pregnancy screening criteria, confirming a gestation period of 28–30 weeks, possessed a mobile phone and expressed willingness to partake in the follow-up study were considered eligible and subsequently enrolled at baseline. Mothers who had actively participated in the baseline study, engaged in the intervention, delivered their babies and were subsequently followed up in both the intervention and control groups for a period extending to 6-weeks postpartum were included in the study (Figure 1).

### Sample size determination and sampling procedures

The study used structural equation modelling (SEM) analysis, which necessitates a specific sample size calculation. We utilized Soper's free statistical a priori sample size calculator for SEM.^32^ This calculator determines the required sample size based on the number of observed and latent variables in the model, the anticipated effect size and the desired probability and statistical power levels. It provides the minimum sample size needed to detect the specified effect and account for the model's structural complexity. Considering an anticipated effect size of 0.3 (medium), a desired statistical power level of 0.8, five latent variables (wealth index, women's autonomy, knowledge, attitude and neonatal care practice), 53 observed variables in the model, a type I error rate of 0.05 and a 10% non-response rate, the final sample size was determined to be 784.

A total of 784 participants (392 intervention and 392 control) were recruited from randomly selected clusters in the study area. The study employed a cluster randomized controlled trial behavioural intervention. Initially, 39 clusters with homogeneous characteristics were identified and 20 clusters were randomly selected from this pool. A census was conducted to identify eligible pregnant women from selected clusters and all eligible pregnant women were included in the selected clusters. Each cluster varies in size, with some clusters having a larger population than others. To account for these size differences and ensure a representative sample, we used a probability proportional to size (PPS) cluster selection method.

### Study variables

In a multivariate SEM analysis, variables were classified into four categories: endogenous, exogenous, latent and observed.^32^

#### Endogenous variables

- Latent variables: neonatal care practices, which is the outcome variable, knowledge and attitude.
- Observed variables: all item constructs or indicators that were used to measure neonatal care practice.

#### Exogenous variables

- Latent variables: wealth index, knowledge attitude and women's autonomy.
- Observed variables: mobile health intervention, age, marital status, age at marriage, sex of newborn, education, occupation, family size, parity, birth interval, complication during delivery and health insurance.

### Description of the intervention

The mHealth intervention aimed to improve neonatal care practices among mothers through behaviour change strategies designed to enhance maternal and child health outcomes by promoting community-based neonatal care. Participants were initially divided into two groups via cluster randomization. The intervention group received SMS messages with healthcare practices over a 4-month period (90 d prenatal and 42 d postpartum). In contrast, the control group continued with standard healthcare practices as part of routine health facility activities. Each participant in the intervention group received one mobile text message every 2 weeks, for a total of eight SMS messages over the 4-month period.

### Intervention module development and components

The mHealth messages for the neonatal care intervention were developed by researchers using culturally appropriate guidelines from national and WHO essential neonatal care references.^2,29,30^ Participants in the intervention group received mHealth education messages covering community-based neonatal care practices. These included congratulations, guidance on maternal and neonatal health, antenatal care, skilled delivery, breastfeeding, thermal care, bathing, cord care, advice against traditional medicine and future pregnancy planning. Experts in behaviour change and neonatal care reviewed and refined the messages. Initially created in English, the messages were then translated into Amharic. Details of the intervention schedule and message content are provided in the supplementary material.

### Recruitment and participant timeline

Pregnant women between 26 and 28 weeks of gestation, as per WHO eligibility criteria, were recruited for the study. Participants were required to provide informed consent, have access to a mobile phone, agree to follow-up and be willing to receive health messages. Eligible participants underwent a baseline assessment. The intervention began at 30 weeks of gestation and continued through postpartum week 6. The research team monitored message delivery, tracked intervention completion and identified dropouts. End-line data were then collected from both the intervention and control groups.

### Randomization to intervention or control group assignment

To prevent contamination, a 30-km buffer zone was established between Dessie and Kombolcha cities. Clusters were defined by kebeles in these cities. After baseline data collection, clusters were allocated to the intervention or control group using stratified randomization based on the average number of pregnant women served per month and geographic location. Clusters within each stratum were randomly assigned to groups using a computer-generated sequence to ensure unbiased randomization and minimize confounding factors. This process aimed to balance the distribution of clusters and enhance the study's reliability and validity. Outcome assessors were blinded to group allocation to prevent bias.

### Strategies to maintain intervention fidelity

To maintain intervention fidelity, several strategies were employed: comprehensive training sessions for those delivering the intervention, use of training manuals and protocols, development of scripts and timing guidelines and instructions for addressing common issues. Additionally, supervision and monitoring visits were conducted to observe the delivery of the intervention and provide feedback.

### Assessment methods to ensure the intended content reached the target audience

The investigators used several assessment techniques to ensure the intended content reached the target audience. The mHealth platform employed in the study included mechanisms to confirm message delivery (sent, delivered) to participants’ mobile phones. Follow-up surveys were conducted to collect feedback on participants’ interactions and understanding of the intervention messages during the end-line evaluation. Self-report measures from implementers were used to assess their adherence to intervention procedures and to identify any challenges encountered.

### Data collection procedures and quality control

Eight trained nurses, familiar with the local geography and fluent in Amharic, were recruited for participant recruitment and baseline data collection. Four Master of Public Health (MPH) holders supervised the data collection process alongside the investigators. Before data collection, a census was conducted to determine eligible pregnant women, including their contact details and follow-up addresses. Each registered woman was assigned an identification number to ensure anonymity and facilitate data linkage. Baseline data were collected through home visits, and end-line surveys were conducted door-to-door 60 d postpartum. To ensure data quality and reliability, training sessions and a pretest of the tool were performed for data collectors and supervisors.

### Data analysis procedures and model assumption

Data collected via ODK Collect were exported to SPSS version 26 (IBM, Armonk, NY, USA) for variable coding, transformations and preparation for analysis. Descriptive statistics, including proportions, percentages, means and measures of dispersion, were computed using tables and graphs. SEM was conducted using Stata version 17 (StataCorp, College Station, TX, USA) to evaluate the impact of the mobile health intervention on neonatal care practices. A measurement model was developed to assess whether observed variables reliably reflected latent variables, with confirmatory factor analysis (CFA) used to test construct validity, including convergent and discriminant validity. After confirming the measurement model, the structural model was created to examine the outcome variable. Model fit was assessed using indices: a χ^2^:degrees of freedom ratio of ≤5, root mean square error of approximation values <0.06 and standardized root mean square residual values <0.08, indicating good fit. Statistical significance was set at a p-value <0.05 with a 95% confidence interval (CI). Model assumptions, including multivariate normality and sample size adequacy, were checked. The model was properly specified with positive degrees of freedom and multicollinearity was assessed through a correlation matrix, where all correlations were <0.8.

### Operational definitions

#### Neonatal care practice

The minimum neonatal care package was adapted from the WHO, with 12 items used to produce a composite index (score) by using CFA. The 12 items were measured in ‘yes’ or ‘no’ responses. Yes was given a value of 1 and no was given a value of 0. CFA was done to create a composite index (score) and respondents who scored above or equal to the median value were considered to have good neonatal care, while those who scored below the median value were considered to have poor neonatal care practice.^2^

#### Knowledge of neonatal care

Twelve questions were used to measure the knowledge of the respondents on neonatal care. If respondents got the right answer, it was coded as 1 (yes), if not, it was coded as 0 (no). The knowledge score was developed through principal component analysis and the knowledge index was developed and treated as a continuous variable.^8^

#### mHealth

mHealth refers to the employment of wireless, moveable data and communication technologies to support health and healthcare. For this study, mHealth includes SMS on neonatal care for behaviour change interventions and reminders.^28^

#### Attitude

Ten questions with a 5-point Likert scale were used to measure the attitude of women towards neonatal care. Respondents who scored above or equal to the median value were considered to have a positive attitude and those who scored below the median value were considered to have a negative attitude.^29^

#### Women’s autonomy

We used 23 items for the categories decision-making autonomy, movement autonomy and financial autonomy. Those who scored above the median were considered autonomous.^30^

### Ethical issues

This study adhered to the Helsinki Declaration and received ethical approval from the Ethical Review Committee of Jimma University, Institute of Health (reference JUIH/IRB 229/22). Written permissions were obtained from relevant authorities in Dessie and Kombolcha. After approval, the principal investigator acquired three SIM cards from Ethio Telecom for the SMS-based intervention. Participants were informed about the study's aims, their rights and the importance of their participation, and written informed consent was obtained. Participation was voluntary and participants could withdraw at any time. For those unable to read the messages, arrangements were made to have a trusted family member or husband read them. If participants did not own a mobile phone but a household member did, they were linked with the mobile owner. Interviews were conducted privately and no personal identifiers were recorded, to maintain confidentiality and anonymity.

## Results

### Sociodemographic and economic characteristics of the participants

The study revealed similar age distributions in both groups, with most participants 25–34 y of age. Housewives were the largest occupational group in both, comprising 50.4% of the control group and 51.9% of the intervention group. More women in the intervention group had a primary education (42.6%) compared with the control group (28.6%). Conversely, the control group had higher percentages with secondary (43.6% vs 39.4%) and higher education (21.3% vs 13.6%) (Table 1).

**Table 1.** Sociodemographic characteristics of study participants on the effectiveness of mHealth interventions to enhance neonatal care practice in northeast Ethiopia, 2023 (N=743)

Variables	Categories	Study group	
		Control, n (%)	Intervention, n (%)
Age (years)	18–24	47 (12.8)	48 (12.8)
	25–29	133 (36.2)	148 (39.4)
	30–34	117 (31.9)	131 (34.8)
	≥35	70 (19.1)	49 (13.0)
	Total	367 (100.0)	376 (100.0)
Occupation	Housewife	185 (50.4)	195 (51.9)
	Merchant	66 (18.0)	54 (14.4)
	Government employee	78 (21.3)	65 (17.3)
	Private employee	35 (9.5)	58 (15.4)
Religion	Muslim	245 (66.8)	225 (59.8)
	Christian	122 (33.2)	151 (40.2)
Marital status	Married	353 (96.2)	362 (96.3)
	Others	28 (7.6)	21 (5.6)
Women’s education status	Read and write	24 (6.5)	17 (4.5)
	Primary	105 (28.6)	160 (42.6)
	Secondary	160 (43.6)	148 (39.4)
	College and above	78 (21.3)	51 (13.6)
Husbands’ education status	Read and write	20 (5.4)	9 (2.4)
	Primary	60 (16.3)	92 (24.5)
	Secondary	167 (45.5)	178 (47.3)
	College and above	120 (32.7)	97 (25.8)
Marital age (years)	15–17	14 (3.8)	17 (4.5)
	18–24	316 (86.1)	302 (80.3)
	≥25	37 (10.1)	57 (15.2)
Family size	≤4	274 (74.7)	255 (67.8)
	>4	93 (25.3)	121 (32.2)
Wealth index	Poor	142 (38.7)	163 (43.4)
	Middle	58 (15.8)	48 (12.8)
	Rich	167 (45.5)	165 (43.9)
Have health insurance	No	245 (66.8)	259 (68.9)
	Yes	136 (37.1)	124 (33.0)
Woman’s autonomy	Not autonomous	207 (56.4)	195 (51.9)
	Autonomous	160 (43.6)	181 (48.1)
Sex of newborn	Male	188 (51.2)	178 (47.3)
	Female	193 (52.6)	205 (54.5)

### Obstetrics characteristics of the participants

The study found that birth-to-pregnancy intervals were similar, with 48.5% in the control and 49.5% in the intervention group having intervals <24 months. Most rated their proximity to health facilities as medium (61.9% control, 71.3% intervention). Health centre deliveries were common (56.4% control, 53.7% intervention), but hospital deliveries were more frequent in the intervention group (33.8%) compared with the control group (16.9%). Normal deliveries were predominant (90.7% control, 87.2% intervention), while operative/Caesarean section rates were slightly higher in the intervention group (12.8% vs 9.3% in the control group) (Table 2).

**Table 2.** Obstetric characteristics of study participants on effectiveness of mHealth intervention to enhance neonatal care practice in Dessie and Kombolcha, northeast Ethiopia, 2023 (N=743)

Variables	Categories	Study group	
		Control, n (%)	Intervention, n (%)
Parity	Primipara	56 (15.3)	44 (11.7)
	Multipara	311 (84.7)	332 (88.3)
Birth to pregnancy interval (months)	<24	178 (48.5)	186 (49.5)
	≥24	189 (51.5)	190 (50.5)
Had ANC follow-up	No	35 (9.5)	48 (12.8)
	Yes	332 (90.5)	328 (87.2)
Distance of health facility from home	Near	93 (25.3)	68 (18.1)
	Medium	227 (61.9)	268 (71.3)
	Far	47 (12.8)	40 (10.6)
Place of delivery	Health centre	207 (56.4)	202 (53.7)
	Home	15 (4.1)	2 (0.5)
	Hospital	62 (16.9)	127 (33.8)
	Private facility	94 (25.6)	52 (13.8)
Mode of delivery	Operative	34 (9.3)	48 (12.8)
	Normal delivery	333 (90.7)	328 (87.2)
Accompanied by social support during delivery	Husband	142 (38.6)	136 (36.3)
	Mother	116 (31.6)	86 (22.8)
	Other family	97 (26.4)	104 (27.6)
	None	12 (3.4)	8 (2.3)
Gestational age at delivery (weeks)	<37	12 (3.3)	40 (10.6)
	≥37	355 (96.7)	336 (89.4)
Complication during delivery	No	307 (83.7)	273 (72.6)
	Yes	60 (16.3)	103 (27.4)
Future fertility preference	Want no more child	36 (9.8)	45 (12.0)
	Undecided	44 (12.0)	83 (22.1)
	Want more, but need time not decide	110 (30.0)	107 (28.5)
	Want later	156 (42.5)	120 (31.9)
	Want more soon	21 (5.7)	21 (5.6)

### Knowledge about neonatal care

The study revealed awareness of neonatal care increased markedly in the intervention group, increasing from 62.0% to 85.9%, compared with a more modest increase in the control group from 57.8% to 67.6%. Improvements in recognizing symptoms of neonatal eye infections were notable in the intervention group, with correct responses increasing significantly for redness (from 27.7% to 67.3%) and discharge (from 28.2% to 56.6%) (Table 3).

**Table 3.** Knowledge about neonatal care of study participants on effectiveness of mHealth intervention to enhance neonatal care practice in Dessie and Kombolcha, northeast Ethiopia, 2023 (N=743)

Variables	Categories	Study group			
		Control, n (%)		Intervention, n (%)	
		Baseline	Endline	Baseline	Endline
Heard about neonatal care	No	155 (42.2)	128 (34.9)	143 (38.0)	44 (11.7)
	Yes	212 (57.8)	248 (67.6)	233 (62.0)	323 (85.9)
Newborn can warm with clean cloth immediately	No	93 (25.3)	178 (48.5)	171 (45.5)	160 (42.6)
	Yes	274 (74.7)	189 (51.5)	205 (54.5)	216 (57.4)
After cutting the umbilical cord, uncover it and keep it dry and clean	No	44 (12.0)	182 (49.6)	128 (34.0)	141 (37.5)
	Yes	323 (88.0)	185 (50.4)	248 (66.0)	235 (62.5)
After cutting the umbilical cord cover the stump with a cloth	No	242 (65.9)	178 (48.5)	284 (75.5)	160 (42.6)
	Yes	125 (34.1)	189 (51.5)	92 (24.5)	216 (57.4)
Sign of neonatal eye infection: redness	No	161 (43.9)	203 (55.3)	272 (72.3)	123 (32.7)
	Yes	206 (56.1)	164 (44.7)	104 (27.7)	253 (67.3)
Sign of neonatal eye infection: discharge	No	256 (69.8)	104 (28.3)	270 (71.8)	163 (43.4)
	Yes	111 (30.2)	263 (71.7)	106 (28.2)	213 (56.6)
Sign of neonatal eye infection: eyelid swelling	No	279 (76.0)	201 (54.8)	245 (65.2)	272 (72.3)
	Yes	88 (24.0)	166 (45.2)	131 (34.8)	104 (27.7)
Sign of umbilical cord infection: foul smell	No	136 (37.1)	235 (64.0)	241 (64.1)	235 (62.5)
	Yes	231 (62.9)	132 (36.0)	135 (35.9)	141 (37.5)
Sign of umbilical cord infection: discharge	No	103 (28.1)	82 (22.3)	183 (48.7)	160 (42.6)
	Yes	264 (71.9)	285 (77.7)	193 (51.3)	216 (57.4)
Sign of umbilical cord infection: redness	No	155 (42.2)	170 (46.3)	143 (38.0)	204 (54.3)
	Yes	212 (57.8)	197 (53.7)	233 (62.0)	172 (45.7)
Sign of umbilical cord infection: area swelling	No	65 (17.7)	211 (57.5)	96 (25.5)	269 (71.5)
	Yes	302 (82.3)	156 (42.5)	280 (74.5)	107 (28.5)
Colostrum should be removed before giving breast milk to the newborn	No	279 (76.0)	279 (76.0)	245 (65.2)	245 (65.2)
	Yes	88 (24.0)	88 (24.0)	131 (34.8)	131 (34.8)
Clean the eyes of the newborn	Upper to lower	166 (45.2)	132 (36.0)	283 (75.3)	183 (48.7)
	Inner to outer	201 (54.8)	235 (64.0)	93 (24.7)	193 (51.3)
Dung can be applied to the umbilical cord	No	314 (85.6)	343 (93.5)	329 (87.5)	352 (93.6)
	Yes	53 (14.4)	24 (6.5)	47 (12.5)	24 (6.4)
Time to bathe newborn	<24 h	178 (48.5)	235 (64.0)	251 (66.8)	162 (43.1)
	>24 h	189 (51.5)	132 (36.0)	125 (33.2)	214 (56.9)
Warm newborn by skin-to-skin contact	No	119 (32.4)	241 (65.7)	235 (62.5)	149 (39.6)
	Yes	248 (67.6)	126 (34.3)	141 (37.5)	227 (60.4)
Need for prelacteal feeding before breast milk to the newborn	No	256 (69.8)	104 (28.3)	270 (71.8)	163 (43.4)
	Yes	111 (30.2)	263 (71.7)	106 (28.2)	213 (56.6)
Time to start/initiate breastfeeding after child birth	After 1 h	65 (17.7)	160 (43.6)	96 (25.5)	82 (21.8)
	With in 1 h	302 (82.3)	216 (58.9)	280 (74.5)	285 (75.8)

### Attitude towards neonatal care

The study compared beliefs about neonatal care between the control and intervention groups, revealing significant differences. The intervention group showed stronger recognition (91.8%) of the benefits of skin-to-skin contact compared with the control group (85.6%). Disagreement regarding immediate newborn bathing was more prevalent in the intervention group (73.9%) than in the control group (58.9%). Preparation of neonate clothes before birth was more prevalent in the intervention group (85.1%) than the control group (76.3%). Finally, belief in the usefulness of colostrum was markedly higher in the intervention group (84.6%) compared with the control group (70.0%) (Table 4).

**Table 4.** Attitudes towards neonatal care of study participants on effectiveness of mHealth intervention to enhance neonatal care practice in Dessie and Kombolcha, northeast Ethiopia, 2023 (N=743)

Variables	Categories	Study group	
		Control, n (%)	Intervention, n (%)
Belief that a newborn baby can be bathed immediately after birth	Disagree	14 (3.8)	40 (10.6)
	Agree	353 (96.2)	336 (89.4)
Belief in importance of prelacteal feeding	Disagree	53 (14.4)	31 (8.2)
	Agree	314 (85.6)	345 (91.8)
Perception of the importance of exclusive breastfeeding for the first 6 months	Disagree	216 (58.9)	278 (73.9)
	Agree	151 (41.1)	98 (26.1)
Need for regular postnatal check-ups for newborns	Disagree	329 (89.6)	349 (92.8)
	Agree	38 (10.4)	27 (7.2)
Perception of the importance of maintaining hygiene in neonatal care	Disagree	67 (18.3)	44 (11.7)
	Agree	300 (81.7)	332 (88.3)
Perception that neonatal care starts before pregnancy	Disagree	317 (86.4)	338 (89.9)
	Agree	50 (13.6)	38 (10.1)
Exclusive breastfeeding for the first 6 months	Disagree	131 (35.7)	122 (32.4)
	Agree	236 (64.3)	254 (67.6)
Perception of the importance of exclusive breastfeeding for the first 6 months	Disagree	87 (23.7)	56 (14.9)
	Agree	280 (76.3)	320 (85.1)
Belief in the benefits of skin-to-skin contact immediately after birth	Disagree	110 (30.0)	58 (15.4)
	Agree	257 (70.0)	318 (84.6)

### Neonatal care practice

**Figure 2.**
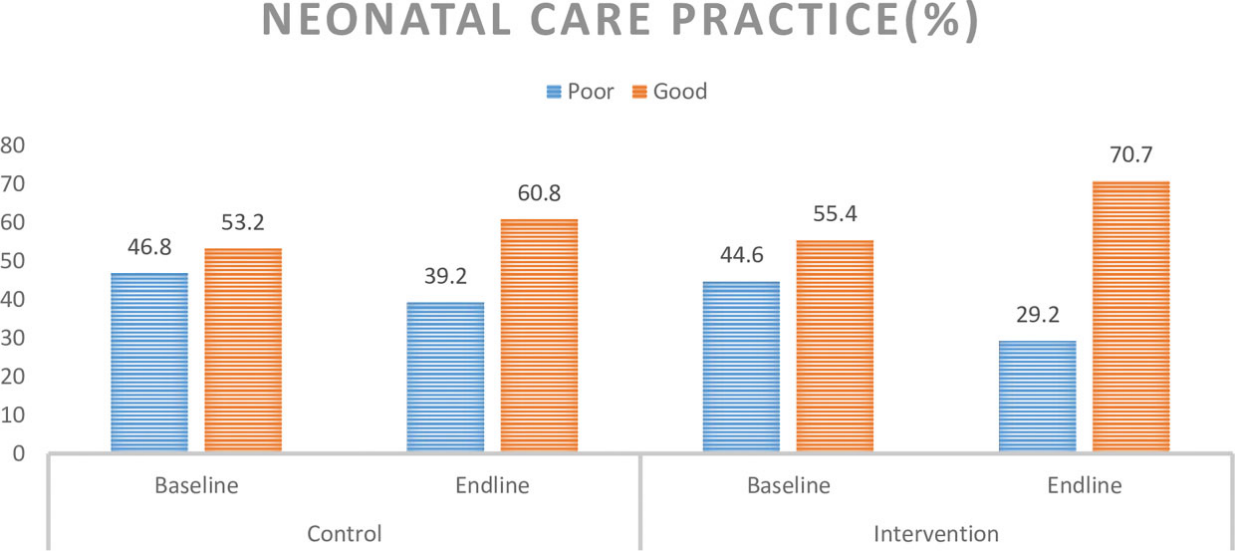
Neonatal care practice of participants from the study on impact of mHealth intervention to enhance neonatal care practice in northeast Ethiopia, 2023.

Delaying newborn bathing for >24 h was more common in the intervention group (64.4%) than in the control group (59.7%). A significant majority practiced feeding colostrum to newborns, with 93.4% in the intervention group and 80.1% in the control group. Initiating breastfeeding within 1 h after birth was higher in the intervention group (85.4%) compared with the control group (74.4%). Complementary feeding for newborns started in 34.6% of the intervention group and 30.0% of the control group. Postnatal visits to health facilities were more frequent in the intervention group (79.6%) than in the control group (54.8%). Prelacteal feeding rates were slightly lower in the intervention group (69.5%) compared with the control group (73.7%). Skin-to-skin contact with the mother after birth was more prevalent in the intervention group (73.9%) compared with the control group (67.3%) (Table 5, Figure 2).

**Table 5.** Neonatal care practice of study participants on effectiveness of mHealth intervention to enhance neonatal care practice in northeast Ethiopia, 2023 (N=743)

Variables	Categories	Study group	
		Control, n (%)	Intervention, n (%)
Antenatal care during the pregnancy	No	48 (12.8)	35 (9.5)
	Yes	328 (87.2)	332 (90.5)
Dung applied on the umbilical cord	No	356 (97.0)	367 (97.6)
	Yes	11 (3.0)	9 (2.4)
Baby bathed after 24 h	No	148 (40.3)	134 (35.6)
	Yes	219 (59.7)	242 (64.4)
Baby fed colostrum	No	73 (19.9)	25 (6.6)
	Yes	294 (80.1)	351 (93.4)
Initiated breastfeeding within 1 hr after birth	No	94 (25.6)	55 (14.6)
	Yes	273 (74.4)	321 (85.4)
Exclusive breastfeeding	No	95 (25.9)	87 (23.1)
	Yes	272 (74.1)	289 (76.9)
Complimentary feeding started for the newborn	No	257 (70.0)	246 (65.4)
	Yes	110 (30.0)	130 (34.6)
Postnatal visit to a health facility to check the health status of the baby	No	75 (20.4)	170 (45.2)
	Yes	292 (79.6)	206 (54.8)
Newborn received any immunization after birth	No	24 (6.5)	16 (4.3)
	Yes	343 (93.5)	360 (95.7)
Prelacteal feeding is given to the baby	No	112 (30.5)	99 (26.3)
	Yes	255 (69.5)	277 (73.7)
Health facility delivery	No	15 (4.1)	2 (0.5)
	Yes	352 (95.9)	374 (99.5)
Skin-to-skin contact with the mother after birth	No	120 (32.7)	98 (26.1)
	Yes	247 (67.3)	278 (73.9)

### Impact of the interactive mHealth intervention based on the SEM analysis

The analysis using SEM was conducted in a two-step process. Initially, the measurement model was assessed through CFA to evaluate the construct validity of the tool. Subsequently, the model, which included the mHealth intervention and modifying variables, was executed to confirm relationships and associations among exogenous and endogenous variables.

### Measurement of SEM

The Kaiser–Meyer–Olkin test confirmed sample adequacy for factor analysis, while Bartlett's test (p=0.000) showed the correlation matrix was not an identity matrix. CFA revealed all factor loadings were >0.5 with p-values <0.05 and average variance extracted (AVE) for all constructs was >0.5, indicating good convergent validity. Discriminant validity was also supported, as the square root of the AVE for each construct was greater than its correlations with other constructs. The tool demonstrated strong construct validity and internal consistency, with Cronbach's α values >0.7 for all scales.

### CFA for knowledge on neonatal care practice measurement

The measurement model for knowledge of neonatal care practices among postpartum women in northeast Ethiopia demonstrated that all items have significant positive factor loadings on the latent construct. The coefficients range from 0.582448 to 6.701315, indicating varying levels of contribution to the construct. The highest contributing items—clean the eyes of the newborn and time of the bath of the newborn—have exceptionally high coefficients, suggesting they are key indicators of neonatal care knowledge. All factor loadings are statistically significant (p<0.0001), reinforcing the reliability of these items in measuring the knowledge construct (Table 6).

**Table 6.** Results of standardized factor loadings of a measurement model for knowledge of neonatal care practice among postpartum women, northeast Ethiopia

Measurement	β coefficient	Standard error	Z	p-Value	95% CI
Newborn warm	1.516 512	0.0 730 518	20.76	0.000	1.3733 to 1.659 691
Uncover umbilical cord/dry	1.197 019	0.0 605 594	19.77	0.000	1.078325 to 1.315 713
Hygiene to prevent infection	1.060 681	0.0 599 543	17.69	0.000	0.9 431 728 to 1.178 189
Neonatal eye infection	1.18 014	0.0 599 262	19.69	0.000	1.062 694 to 1.2976
Newborn immunization	1.400 158	0.0 656 989	21.31	0.00	1.27 139 to 1.528 925
Exclusive breastfeeding	0.8 693 871	0.0 485 503	17.91	0.000	0.7 742 302 to 0.9 645 439
Neonatal care practices	0.842 537	0.0 482 979	17.44	0.000	0.7 478 756 to 0.93 719
Regular postnatal check-ups	1.452 361	0.0 670 406	21.66	0.000	1.3209 to 1.5837
Benefits of skin-to-skin contact	1.132 996	0.0 569 473	19.90	0.000	1.021 381 to 1.24 461
Umbilical cord infection	0.8 108 055	0.0 479 775	16.90	0.000	0.7 167 714 to 0.9 048 397
Colostrum feeding	0.5 824 476	0.0 435 739	13.3	0.000	0.4 970 443 to 0.6 678 509
Clean the eyes of the newborn	3.538 554	0.1 678 725	21.08	0.000	3.20 953 to 3.867 578
Time to bathe newborn	6.701 315	0.3 339 058	20.07	0.000	6.046 872 to 7.355 758

### CFA for attitude towards neonatal care measurement

The measurement model for attitudes towards neonatal care practices among postpartum women in northeast Ethiopia showed all items have significant positive factor loadings on the latent construct, with coefficients ranging from 1.081 to 2.606. The highest contributing items are beliefs that a newborn can be bathed immediately after birth, neonatal care starts before pregnancy and the importance of prelacteal feeding, indicating they are key indicators of attitudes towards neonatal care practices. All factor loadings are statistically significant (p<0.0001), demonstrating the reliability of these items in measuring the attitude construct (Table 7).

**Table 7.** Results of standardized factor loadings of a measurement model for attitude towards neonatal care practice in postpartum women, northeast Ethiopia

Measurement	β coefficient	Standard error	Z	p-Value	95% CI
Exclusive breastfeeding	1.080 652	0.0 272 418	39.67	0.000	1.027 259 to 1.134 045
Postnatal check-ups for newborns	2.155 462	0.0 555 881	38.78	0.000	2.046 511 to 2.264 413
Belief on newborn bath	2.60 604	0.0 588 464	44.29	0.000	2.490 703 to 2.721 377
Importance of prelacteal feed	1.175 372	0.0 304 616	38.59	0.000	1.115 668 to 1.235 075
Maintaining hygiene in neonatal care	2.478 749	0.0 584 321	42.42	0.000	2.364 225 to 2.593 274
Skin-to-skin contact after birth	1.409 895	0.0 358 158	39.37	0.000	1.339 698 to 1.480 093
Neonatal care starts before pregnancy	1.188 851	0.0 292 738	40.61	0.000	1.131 476 to 1.246 227
Prepare cloth before birth	1.216 271	0.0 317 085	38.36	0.000	1.154 123 to 1.278 418

### CFA for neonatal care practice measurement

The measurement model for neonatal care practices among postpartum women in northeast Ethiopia showed all items have significant positive factor loadings on the latent construct, with coefficients ranging from 0.696 to 1.092. The highest contributing items are immunization and skilled birth attendant, indicating they are key indicators of neonatal care practices. All factor loadings are statistically significant (p<0.0001), demonstrating the reliability of these items in measuring neonatal care practices (Table 8). The results emphasize that each item significantly contributes to the overall understanding of neonatal care practices in this region.

**Table 8.** Results of standardized factor loadings of a measurement model for neonatal care practice in postpartum women, northeast Ethiopia

Measurement	β coefficient	Standard error	Z	p-Value	95% CI
Antenatal care	0.8 882 907	0.0 115 564	76.87	0.000	0.8 656 406 to 0.9 109 409
Bath	0.6 961 629	0.0 218 075	31.92	0.000	0.6 534 209 to 0.7 389 049
Colostrum feeding	0.9 763 321	0.0 182 819	53.40	0.000	0.9 405 003 to 1.012 164
Breastfeeding initiation	0.8 985 408	0.0 198 266	45.32	0.000	0.8 596 814 to 0.9 374 002
Exclusive breastfeeding	0.8 474 237	0.0 206 042	41.13	0.000	0.8 070 402 to 0.8 878 073
Postnatal care	0.7 630 945	0.0 210 171	36.31	0.000	0.7219018 to 0.8 042 871
Immunization	1.060 612	0.0 161 352	65.73	0.000	1.028 988 to 1.092 236
Prelacteal feeding	0.8 032 987	0.0 210 995	38.07	0.000	0.7 619 444 to 0.8 446 531
Skilled birth attendant	1.091 846	0.015 254	71.58	0.000	1.061 949 to 1.121 744
Skin-to-skin contact	0.7 932 574	0.0 211 776	37.46	0.000	0.7 517 499 to 0.8 347 648

### Direct, indirect and total effects of mHealth intervention and modifying factors on neonatal care practice

**Figure 3.**
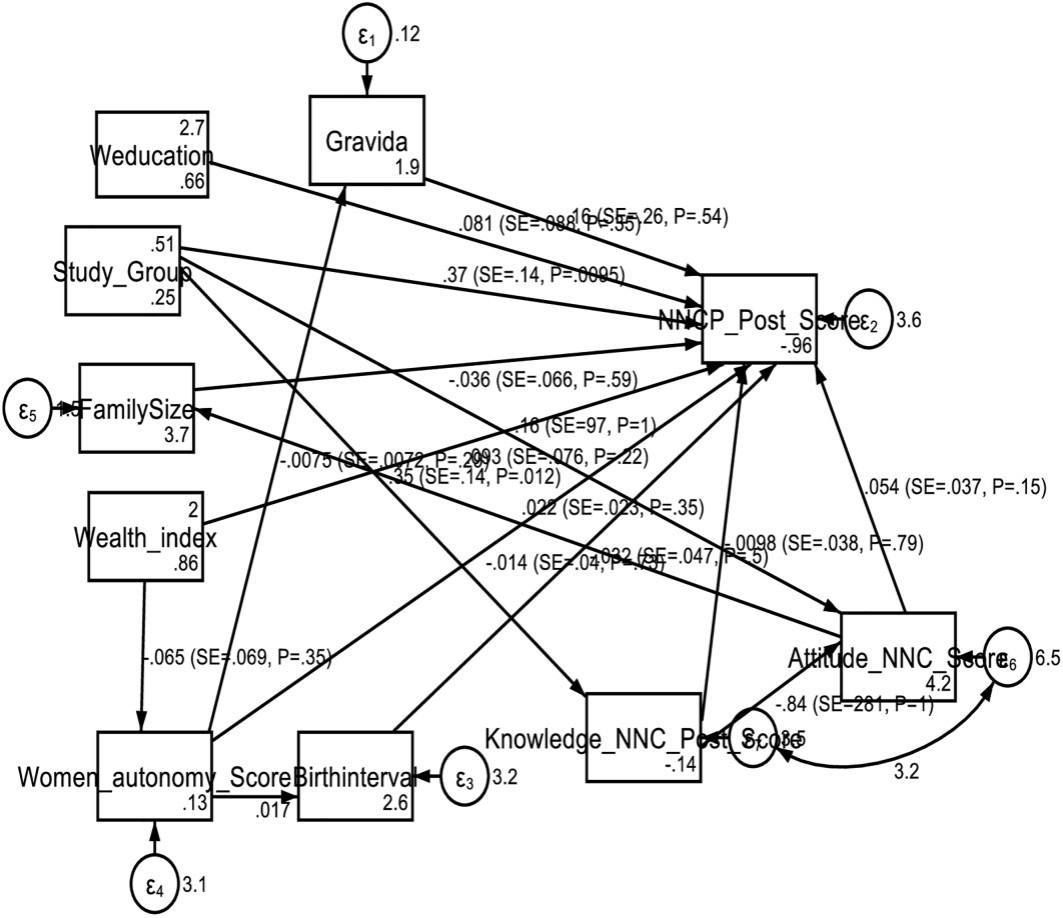
SEM (path analysis) with standardized coefficients of neonatal care practice among postpartum mothers in Dessie and Kombolcha, northeast Ethiopia.

The mHealth intervention has a significant positive effect on neonatal care practices (β=0.393, p=0.007), enhancing the care practices among postpartum women. Increased knowledge of neonatal care also positively impacts care practices (β=0.347, p=0.012). However, attitudes towards neonatal care (β=0.047, p=0.212) and other factors such as family size, women's education, paid work, autonomy, age, fertility preference, wealth index and birth interval do not significantly impact neonatal care practices. No significant indirect pathways were found between the variables. The total effects, combining direct and indirect effects, reaffirm that the mHealth intervention (β=0.382, p=0.009) and knowledge of neonatal care (β=0.347, p=0.012) are significant predictors of improved neonatal care practices. Other factors do not show substantial impacts. The SEM highlights the crucial role of the mHealth intervention and knowledge in improving neonatal care practices. The intervention increases the care practice score by 0.393 units per unit of engagement, and knowledge increases the score by 0.347 units per unit of knowledge (Table 9, Figure 3).

**Table 9.** Direct, indirect and total effects of mHealth interventions and modifying factors on neonatal care practice among postpartum women, northeast Ethiopia

Variables pathway	Direct effect, β (SE), p-value	Indirect effect, β (SE), p-value	Total effect, β (SE), p-value
Attitude towards neonatal care	0.0466 (0.0373), 0.212	0 (no path)	0.0466 (0.0373), 0.212
Family size	−0.0660 (0.0749), 0.378	0 (no path)	−0.0660 (0.0749), 0.378
Women education	0.0695 (0.0876), 0.428	0 (no path)	0.0695 (0.0876), 0.428
Women have paid work	−0.1871 (0.1598), 0.242	0 (no path)	−0.1871 (0.1598), 0.242
Women autonomy	−0.0153 (0.0402), 0.704	0 (no path)	−0.0153 (0.0402), 0.704
mHealth intervention	0.3931 (0.1464), 0.007	−0.01 (0.015), 0.495	0.3823 (0.1456), 0.009
Mother’s age	0.0227 (0.0204), 0.266	0 (no path)	0.0227 (0.0204), 0.266
Fertility preference	−0.0420 (0.0671), 0.531	0 (no path)	−0.0420 (0.0671), 0.531
Wealth	−0.0199 (0.0381), 0.603	0 (no path)	−0.0199 (0.0381), 0.603
Birth interval	−0.0324 (0.0434), 0.456	0 (no path)	−0.0324 (0.0434), 0.456
Knowledge of neonatal care	0.3467 (0.1375), 0.012	0 (no path)	0.3467 (0.1375), 0.012

## Discussion

The study found that around half of the participants in both intervention and control groups had birth-to-pregnancy intervals <24 months. This finding aligns with other research^33^ that reports high rates of short birth intervals in low-resource settings. Recent studies^34^ stress the necessity of comprehensive family planning programs to extend birth intervals and improve maternal and child health outcomes. This suggests that integrating mHealth interventions with family planning education could effectively address both immediate and long-term health needs.

The study revealed mixed results in umbilical cord care practices. In the intervention group, covering the umbilical stump with cloth significantly decreased (from 75.5% to 42.6%), but improvements in keeping the stump dry and clean were minimal (from 66.0% to 62.5%). Research by Asfaw et al.^35^ highlights the importance of keeping the stump dry to prevent infections, consistent with WHO guidelines.^36^ The discrepancy, where harmful practices decreased but improved cord care did not fully follow, may be due to incomplete adoption or traditional beliefs. This indicates that while the intervention reduced harmful practices, further comprehensive education and reinforcement are needed. Future programs should focus on thorough education and follow-up to enhance both the avoidance of harmful practices and the promotion of proper cord care techniques.

The study showed significant improvement in recognizing neonatal infection signs in the intervention group, particularly symptoms like eye redness (from 27.7% to 67.3%) and eye discharge (from 28.2% to 56.6%). This aligns with recent findings^37^ that highlight the effectiveness of mHealth tools in improving maternal awareness of neonatal infections. Future programs should expand educational efforts to include a wider range of symptoms and provide ongoing support to enhance neonatal care.

The study demonstrated a significant reduction in the perceived need for prelacteal feeding in the intervention group, from 71.8% to 43.4%. This supports recent research^38^ that shows educational interventions can reduce prelacteal feeding and enhance exclusive breastfeeding. However, the reduction was not complete, possibly due to ongoing cultural beliefs or limited intervention reach. This suggests that while the intervention was effective, more efforts are needed to overcome remaining misconceptions.

The study revealed a notable reduction in the harmful practice of applying dung to the umbilical cord, especially in the intervention group, where the prevalence decreased from 12.5% to 6.4%. This aligns with recent research^39^ that showed educational campaigns effectively reduce unsafe umbilical cord care practices. The greater reduction in the intervention group highlights the effectiveness of targeted education, while the continued presence of the practice in the control group suggests more work is needed. This underscores the need for ongoing education and community involvement to eradicate harmful practices completely.

The study showed a notable increase in the proportion of participants in the intervention group who delayed bathing their newborns for >24 h, from 33.2% to 56.9%, whereas the control group saw a decline. This is consistent with recent findings^40^ that highlight delaying the first bath helps regulate the newborn’s body temperature and prevent hypothermia. The improvement in the intervention group likely resulted from targeted educational efforts promoting the benefits of delayed bathing. The decline in the control group may be due to a lack of reinforcement.

The study found that 91.8% of mothers in the intervention group recognized that skin-to-skin contact prevents the baby from getting cold, compared with 85.6% in the control group. This supports recent research^41^ that highlights the benefits of skin-to-skin contact for maintaining body temperature and promoting bonding. The higher recognition in the intervention group suggests that targeted education effectively improved maternal understanding. The discrepancy between groups likely arises from focused educational efforts in the intervention group, while the control group lacked similar reinforcement. Future interventions should continue to emphasize and reinforce the benefits of this practice.

The study found that fewer mothers in the intervention group (26.1%) believed that newborns should be bathed immediately after birth compared with the control group (41.1%). This is consistent with recent research^42^ that underscores the importance of delaying the first bath to maintain newborn thermal regulation and skin integrity.

The study showed a significantly greater number of postnatal visits in the intervention group (79.6%) compared with the control group (54.8%). This aligns with recent research^43^ that found mHealth interventions and targeted education improved postnatal care attendance by raising awareness of the importance of follow-up visits. The higher visit rate in the intervention group suggests the educational and supportive components were effective. The discrepancy likely stems from the intervention group's focused efforts promoting postnatal visits, unlike the control group.

The significant positive effect of the mHealth intervention on neonatal care practice (β=0.3930815, p=0.007) is consistent with studies^44^ that demonstrated mHealth interventions can improve maternal and neonatal health outcomes through enhanced communication and education. These studies similarly reported that mHealth tools facilitate timely access to health information, resulting in better adherence to neonatal care practices. The significant impact of the mHealth intervention on neonatal care practice highlights the potential for scaling up such programs. Health policymakers and practitioners should consider integrating mHealth tools into existing maternal and child health programs to improve neonatal care practices.

The significant influence of knowledge of neonatal care practice (β=0.3467129, p=0.012) corroborates the findings of other research emphasizing the critical role of maternal knowledge in improving neonatal outcomes. For example, a study^45^ found that mothers with higher levels of knowledge about neonatal care were more likely to practice essential neonatal care behaviours. Given the critical role of knowledge in improving neonatal care practice, targeted educational campaigns and training programs for mothers on neonatal care should be prioritized.

However, the lack of significant direct effects for other factors, such as family size, women's education and women's paid work, on neonatal care practice contrasts with some studies that have found these variables to be influential. For instance, researchers^46,47^ reported that maternal education and employment status positively affect neonatal care practices by enhancing mothers’ autonomy and decision-making power. The discrepancy might be attributed to the specific cultural and socio-economic context of the study area in northeast Ethiopia, where traditional practices and community norms may override the individual-level effects of these variables.

The absence of significant effects for attitude towards neonatal care practice (β=0.046553, p=0.212) and other factors like family size and women's education may be due to several contextual reasons. First, attitudes alone may not translate into practice without corresponding knowledge and practical skills. This aligns with the Theory of Planned Behavior,^48^ which suggests that while attitudes are important, actual behaviour change requires perceived behavioural control and intention, which are more directly influenced by knowledge and supportive interventions like mHealth.

### Practical implications

The study highlights the significant benefits of mHealth interventions in enhancing maternal knowledge and practices related to neonatal care. The practical implications are enhanced maternal education, improved healthcare access and policy and program development of interventions with in the health system.

### Limitations of the study

The reliance on self-reported data for maternal and neonatal practices introduces potential biases such as social desirability bias, where participants may report behaviours they perceive as socially acceptable or desirable rather than their actual practices. Other important indicators of maternal and neonatal health outcomes, such as long-term health impacts or health system costs, were not thoroughly assessed.

## Conclusions

The findings consistently demonstrate the substantial positive impact of mHealth interventions on maternal knowledge and neonatal care practices. Enhanced awareness, improved healthcare access and increased adoption of recommended practices among mothers in the intervention group underscore the potential impact of mHealth strategies in mitigating maternal and neonatal health risks. The mHealth intervention has a significant positive effect on neonatal care practices. Knowledge regarding neonatal care was positively associated with better neonatal care practices.

### Recommendations

Policymakers and programmers should prioritize scaling up integration and expansion of mHealth initiatives to strengthen maternal education, facilitate informed decision-making and sustainably improve neonatal health outcomes by leveraging existing mobile networks and partnerships with healthcare providers to ensure broader access to maternal and neonatal health information. Regional health bureaus and non-governmental organizations should implement robust monitoring and evaluation frameworks to assess the long-term impact of mHealth interventions on maternal and neonatal health outcomes to inform program adjustments and improvements. Healthcare workers should continue using mHealth tools to disseminate critical health information on neonatal care practices, such as skin-to-skin contact, proper umbilical cord care and recognition of neonatal infection signs. Future researchers should identify the cost-effectiveness of mHealth interventions, evaluate call and video mHealth interventions and address sociocultural challenges in maternal and neonatal healthcare delivery through targeted interventions.

## Supplementary Material

ihae080_Supplemental_Files

## Data Availability

All data underlying the findings described in this article are freely available within the manuscript itself and can be uploaded as supplementary information.
