# Supplementary material for: The effect of an interactive mobile health intervention to improve community-based essential neonatal care practices among postpartum women in northeast Ethiopia: a cluster randomized controlled trial
Source: Int Health. 2025 Jan 10;17(5):820–35. doi: 10.1093/inthealth/ihae080 (PMC12406790; doi:10.1093/inthealth/ihae080)
Supplement: ihae080_Supplemental_Files [file ihae080_supplemental_files.zip › Ethical approval letter JUIH.pdf]

Jimma University Institute of Health

Institutional Review Board

Ref.No- JUIH/IRB/228/22

Date: 16/11/2022

To: Mr. Nigus Cherie

Subject: Ethical Approval of Research Protocol

The IRB of Institute of Health has reviewed your research *project* ***"EFFECTIVENESS OF INTERACTIVE MOBILE HEALTH INTERVENTION (IMHI) TO IMPROVE EARLY POSTPARTUM MODERN CONTRACEPTIVE METHOD USE AND NEONATAL CARE PRACTICE AMONG POSTPARTUM WOMEN IN DESSIE AND KOMBOLCHA TOWN ZONES, NORTH EAST ETHIOPIA: BEHAVIOURAL CLUSTER RANDOMIZED CONTROL TRIAL".***

Thus, this is to notify that this research protocol has presented to the IRB meets the ethical and scientific standards outlined in national and international guidelines. Hence, we are pleased to inform you that your research protocol is ethically cleared under the following strict conditions:

1. Any significant deviation from the methodological details indicated in the approved protocol must be communicated to the IRB before it has been implemented.
2. Approval shall be only for a period of twelve months. The principal investigator is required to submit an application for the renewal of the ethical approval.
3. The Committee must be notified, in writing, of any alteration to the project including unforeseen events/circumstances that might affect the acceptability of the approved protocol.
4. The Principal researcher is required to immediately notify the committee in the event of any adverse effects on participants or of any unforeseen events that might affect continued ethical acceptability or amendment to the original consent form.
5. The inability of the Principal Researcher to continue in that role or any other change in research personnel involved in the project should be notified to the committee immediately.

The IRB wishes you every success in your research.

Mr. Gizachew Tilahun
Assistant Professor
Chair, JUIH-IRB

Jimma University Institutional Review board
Jimma University Health Institute

E-mail: ethicsjuirb@gmail.com
