# Supplementary material for: The effect of an interactive mobile health intervention to improve community-based essential neonatal care practices among postpartum women in northeast Ethiopia: a cluster randomized controlled trial
Source: Int Health. 2025 Jan 10;17(5):820–35. doi: 10.1093/inthealth/ihae080 (PMC12406790; doi:10.1093/inthealth/ihae080)
Supplement: ihae080_Supplemental_Files [file ihae080_supplemental_files.zip › Protocol Impact of IMHI on NNCP.pdf]

The effectivness of an Interactive Mobile Health Intervention (IMHI) to improve Community-Based Essential Neonatal Care Practices among Postpartum Women in Northeast Ethiopia: A Cluster Randomized Controlled Trial (RCT): Study Protocol

Niguss Cherie[1 2], Muluemebet Abera Wordofa[1], Gurmesa Tura Debelew[1]*

[1] Population and Family Health Department, Faculty of Public Health, Institute of Health, Jimma University, Jimma, Ethiopia, [2]Reproductive and Family Health Department, School of Public Health, College of Medicine and Health Sciences, Wollo University, Dessie, Ethiopia

Email Address:

Niguss Cherie (MPH/RH, Assistant Professor): niguchern@gmail.com

Prof. Muluemebet Abera (Associate professor, PhD): mulu_abera.ts2009@yahoo.com

Prof. Gurmesa Tura (PhD): gurmesatura@gmail.com

***Corresponding author:** Niguss Cherie

[1] Population and Family Health Department, Faculty of Public Health, Institute of Health, Jimma University, Jimma, Ethiopia,[2]Reproductive and Family Health Department, School of Public Health, College of Medicine and Health Sciences, Wollo University, Dessie, Ethiopia

Email- niguchern@gmail.com

Mobile: +251910749743

Abstract

Background: Despite global declines in child mortality rates, Africa's reduction is lagging behind other regions. Neonatal survival remains a key priority in the sustainable development agenda. Promoting neonatal care practices at individual and community levels is essential, and technology-based interventions can effectively reach potential future mothers. However, there is a lack of evidence demonstrating that mobile health interventions improve community-based neonatal care practices in Ethiopia.

Objectives: This study aimed to evaluate the impact of an interactive mobile health intervention on improving community-based essential neonatal care practices among postpartum women in Northeast Ethiopia.

Methods: The study will be conducted in Dessie and Kombolcha city zones, Northeast Ethiopia. A cluster randomized controlled trial will be implemented among 743 participants (376 in the intervention group and 367 in the control group) from January 15 to May 15, 2024. Pregnant women at 30 weeks of gestation in selected clusters will be enrolled and followed up to 45 days post-childbirth. Data will be collected using Open Data Kit (ODK) and analyzed with STATA 17. Structural Equation Modeling (SEM) through confirmatory factor analysis (CFA) will be employed. Model fitness will be evaluated using the chi-square to degree of freedom ratio, root mean square error of approximation (RMSEA), and standardized root mean square residual (SRMR), indicating a good model fit. Statistical significance will be declared at a level below 0.05 with a 95% confidence interval.

Conclusion: This study will underscore the significant role of mobile health interventions and maternal knowledge in enhancing neonatal care practices. These findings will inform the design and implementation of maternal and child health programs, emphasizing the integration of technology and education to improve neonatal outcomes in resource-limited settings. Future efforts will focus on leveraging these insights to develop and implement targeted interventions that utilize mHealth strategies and enhance maternal education, ultimately aiming to improve neonatal care and health outcomes in underserved communities.

Trial Registration: Protocol Registration and Results System (PRS) Clinical Trial Registry, www.ClinicalTrials.gov, ClinicalTrials.gov ID: NCT05666050. Registered on December 23, 2022.

Keywords: Impact, mobile health, neonatal care practice, randomized controlled trial, Northeast Ethiopia.

Introduction

The time of life, the first 28 days is the most crucial time for a child's growth and survival[1,2]. But, one million neonates die annually globally, and ninety-nine percent of those deaths occur in low-income countries[3]. Though child mortality rates are decreasing globally, the African continent is experiencing abundant slower declines than different regions[4]. Most of those deaths can be simply prevented by promotion and providing an acceptable package of neonatal care practices [5,6].

One major target of the Sustainable Development Goals (SDGs) is to end preventable deaths of newborns, with all countries going to decrease neonatal mortality rate to a minimum of 12 per 1000 live births by 2030[7–

9]. The global health actors, together with researchers, policy manufacturers, and program implementers have been finding new data and technologies for neonatal survival for several years[10]. However, the difficulty of neonatal survival remained unfinished agenda and is among the unachieved millennium development goal targets and is known for the SDG priorities[11].

Community-based interventions are necessary for reducing neonatal deaths, even wherever levels of facility deliveries are high[12]. Once delivery, newborns ought to receive immediate newborn care, which includes thermal care (drying and wrapping, skin-to-skin care, delayed bathing), sanitary cord care, colostrum feeding, and early initiation of breastfeeding[13]. These practices need to be promoted at the individual and community levels through a spread of channels together with technology-based interventions to reach new future digital potential mothers[12].

To reduce maternal and child death, the Ethiopian government did several health interventions like coaching midwives, enhancing referral systems, group action health services and implementing packages of the Health Extension Program[13,14]. However, death remains high, even one in every of the highest 10 countries in Africa[15]. Based on the Ethiopian Demographic and Health Survey (EDHS) reports neonatal death has not decreased and stagnant that indicate the need for new approaches to enhance neonatal care intervention and follow at the community level[16–18]. To enhance the survival of neonates, community-based essential newborn care may be a priority intervention technique[19,20].

The growth and access to mobile phones, mobile services, and increase in mobile penetration in developing countries is anticipated to facilitate the employment of mobile health(mHealth) initiatives in resource-restricted settings [21,22]. Extending the reach of the health care system, mHealth is intended to function cue to action to support health care behavior amendment[23]. Major effects of a mobile-health program embody boosting communications between pregnant women and caregivers at different levels, recording their health standing for a time of care, and providing necessary information, and directing to users [24–26].

There is growing evidence showing that mobile health solutions (mHealth) like text electronic communication (SMS) improve health service delivery processes and health outcomes, notably within the areas of treatment adherence, appointment compliance, and patient watching within the developed world[27]. However, no evidence demonstrates the effectiveness of mHealth interventions on key maternal and child health service outcomes, as well as neonatal care practices in Ethiopia. Therefore, we tend to incorporate the essential neonatal care guideline with the mobile short message, with the rationales that facilitate to improve neonatal care applied with a hypothesis that such intervention would be effective in enhancing community-based neonatal care practices. Thus, the target of this study was to intervene in the effectiveness of interactive mobile health intervention to enhance and improve community-based essential neonatal care among postpartum mothers in Dessie and Kombolcha zones, Northeast Ethiopia. Therefore the findings of this study are expected to contribute to the existing knowledge, to contribute to current gaps within the literature and take a look at

whether or not phone-based support to women throughout pregnancy and early postpartum improves neonatal care practice, to understand the possible technology-based interventions for behavior change in the community and act accordingly. Additionally, the findings can be used as baseline information for improving healthcare services in this area to policymakers, program implementers, NGOs, local health planners, and healthcare providers.

Methods and materials

Study area, design, and period

The study will be conducted in the Dessie and Kombolcha city zones in Amhara regional state, Northeast Ethiopia. Dessie is the administrative town of the south Wollo zone, which is situated 401 KM from Addis Ababa to the north. Dessie city is split into 5 sub cities with 22 kebeles and has 2 governmental hospitals and 8 health centers. Based on population projection for 2023 more than 470,000 residents population with an estimated 21, 620 pregnant women in Dessie town. Kombolcha town is 30 km from Dessie city and 375 km from Addis Ababa is an industrial zone and dry port in northeast Ethiopia. There are more than 350,000 resident populations with 16,100 estimated pregnant women in Kombolcha town. It is divided into 5 sub cities with 19 kebeles and has one governmental hospital and five health centers[31] Cluster randomized control trial study will be conducted from January 15 to Jun 15, 2023.

Population and eligibility criteria

The source population for the study will comprise all postpartum women in the designated area. During enrollment, eligibility criteria will be applied, and pregnant women who meet the World Health Organization (WHO) pregnancy screening criteria—confirming a gestation period of 28-30 weeks, possessing a mobile phone, and expressing willingness to participate in the follow-up study—will be considered eligible and subsequently enrolled at baseline. Mothers who actively participate in the baseline study, engage in the intervention, deliver their babies, and are followed up in both the intervention and control groups for a period extending to 6 weeks postpartum will be included in the study.

Sample size determination and sampling procedures

The study will utilize structural equation modeling (SEM) analysis, which will require a specific sample size calculation. We will use Daniel Soper's free statistical priori sample size calculator for SEM [32]. This calculator will determine the required sample size based on the number of observed and latent variables in the model, the anticipated effect size, and the desired probability and statistical power levels. It will provide the minimum sample size needed to detect the specified effect and account for the model's structural complexity. Considering an anticipated effect size of 0.3 (medium), a desired statistical power level of 0.8, five latent variables (wealth index, women's autonomy, knowledge, attitude, and neonatal care practice), 53 observed variables in the model, a type I error rate of 0.05, and a 10% non-response rate, the final sample size will be determined to be 784. A total of 784 participants (392 in the intervention group and 392 in the control group)

will be recruited from randomly selected clusters in the study area. The study will employ a cluster randomized controlled trial behavioral intervention. Initially, 39 clusters with homogeneous characteristics will be identified, and 20 clusters will be randomly selected from this pool. A census will be conducted to identify eligible pregnant women from the selected clusters, and all eligible pregnant women will be included. To account for variations in cluster size and ensure a representative sample, a Probability Proportionate to Size (PPS) cluster selection method will be used.

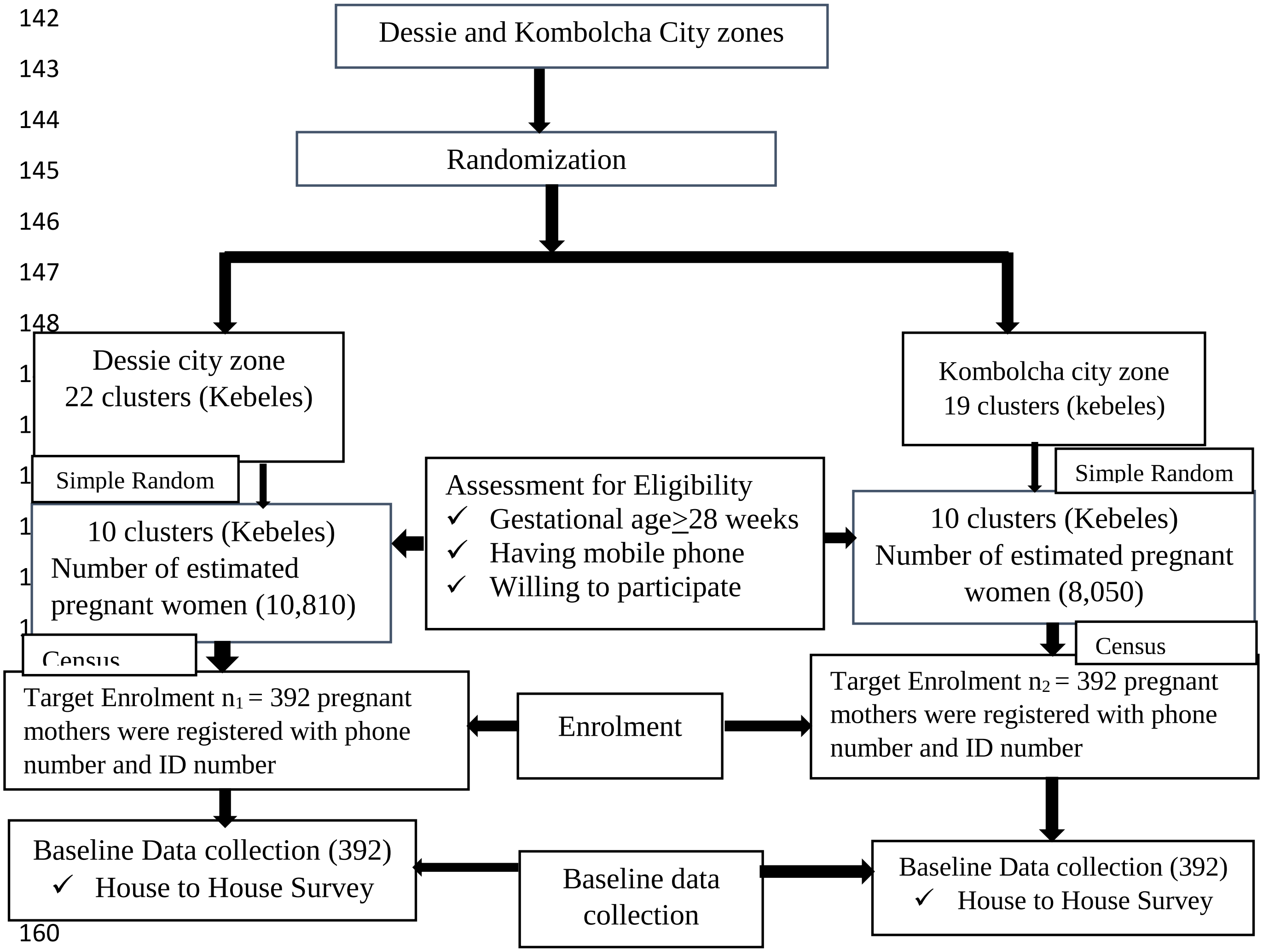

Fig 1: Consolidated Standards of Reporting Trials (CONSORT) Diagram depicts the sampling method and allocation of study units, assessment of eligibility criteria to include study participants to the impact of mobile health intervention to improve community based neonatal care practice in Dessie and Kombolcha city zones, Northeast Ethiopia.

Study variables

In a multivariate Structural Equation Modeling (SEM) analysis, variables will be classified into four categories endogenous, exogenous, latent, and observed variables [32]. In this regard, the variables were classified as follows.

Endogenous variable

Latent variables: Neonatal care practices; which is the outcome variable, Knowledge and attitude will latent endogenous variables.

Observed variables: All item constructs or indicators that were used to measure neonatal care practice will be observed endogenous variables.

Exogenous variables:

Latent variables: Exogenous latent variables will be wealth index, Kowledge, attitude and women's autonomy.

Observed variables: mobile health intervention, age, marital status, age at marriage, sex of newborn, education, occupation, family size, Parity, birth interval, complication during delivery, and health insurance.

Description of the intervention

The mobile health intervention will aim to improve neonatal care practices among mothers through behavior change strategies designed to enhance maternal and child health outcomes by promoting community-based neonatal care. Participants will be initially divided into two groups via cluster randomization. The intervention group will receive SMS messages about existing healthcare practices over a four-month period (90 days prenatal and 42 days postpartum). In contrast, the control group will continue with standard healthcare practices as part of routine health facility activities. Each participant in the intervention group will receive one mobile text message every two weeks, totaling eight SMS messages over the four-month period.

Recruitment and participant timeline

Pregnant women between 26 and 28 weeks of gestation, according to WHO eligibility criteria, will be recruited for the study. Participants will be required to provide informed consent, have access to a mobile phone, agree to follow-up, and be willing to receive health messages. Eligible participants will undergo a baseline assessment. The intervention will begin at 30 weeks of gestation and continue through the 6th week postpartum. The research team will monitor message delivery, track intervention completion, and identify dropouts. End-line data will then be collected from both the intervention and control groups.

Randomization to intervention or control group assignment

To prevent contamination, a 30 km buffer zone will be established between Dessie and Kombolcha cities. Clusters will be defined by kebeles in these cities. After baseline data collection, clusters will be allocated to intervention or control groups using stratified randomization based on the average number of pregnant women served per month and geographic location. Clusters within each stratum will be randomly assigned to groups using a computer-generated sequence to ensure unbiased randomization and minimize confounding factors. This process will aim to balance the distribution of clusters and enhance the study's reliability and validity. Outcome assessors will be blinded to group allocation to prevent bias.

Strategies to mantain intervention fidelity

To maintain intervention fidelity, several strategies will be employed: comprehensive training sessions for those delivering the intervention, use of training manuals and protocols, development of scripts and timing guidelines, and instructions for addressing common issues. Additionally, supervision and monitoring visits will be conducted to observe the delivery of the intervention and provide feedback.

Assessment methods to intended content reached the target audience

The investigators will use several assessment techniques to ensure the intended content reaches the audience. The mobile health (mHealth) platform employed in the study will include mechanisms to confirm message delivery (sent, delivered) to participants' mobile phones. Follow-up surveys will be conducted to collect feedback on participants' interactions and understanding of the intervention messages during the end-line evaluation. Self-report measures from implementers will be used to assess their adherence to intervention procedures and to identify any challenges encountered.

Data collection procedures and quality control

Eight trained nurses, familiar with the local geography and fluent in Amharic, will be recruited for participant recruitment and baseline data collection. Four Master of Public Health (MPH) holders will supervise the data collection process alongside the investigators. Before data collection, a census will be conducted to list eligible pregnant women, including their contact details and follow-up addresses. Each registered woman will be assigned an identification number to ensure anonymity and facilitate data linkage. Baseline data will be collected through home visits, and end-line surveys will be conducted door-to-door 60 days postpartum. To ensure data quality and reliability, training sessions and a pretest of the tool will be performed for data collectors and supervisors.

Data analysis procedures and model assumption

Data collected via ODK Collect will be exported to SPSS version 26 for variable coding, transformations, and preparation for analysis. Descriptive statistics, including proportions, percentages, means, and measures of dispersion, will be computed using tables and graphs. Structural Equation Modeling (SEM) will be conducted using Stata version 17 to evaluate the impact of the mobile health intervention on neonatal care practices. A measurement model will first be developed to assess whether observed variables reliably reflect latent variables, with confirmatory factor analysis (CFA) used to test construct validity, including convergent and discriminant validity. After confirming the measurement model, the structural model will be created to examine the outcome variable. Model fit will be assessed using indices: a chi-square to degrees of freedom ratio of 5 or less, RMSEA values below 0.06, and SRMR values below 0.08, indicating a good fit. Statistical significance will be set at a p-value less than 0.05 with a 95% confidence interval. Model assumptions, including multivariate normality and sample size adequacy, will be checked. The model will be properly specified with positive degrees of freedom, and multicollinearity will be assessed through a correlation matrix.

Operational definitions

Neonatal care practice: The minimum neonatal care package will be adapted from WHO having 12 items used to produce a composite index (score) by using Confirmatory Factor Analysis (CFA). The 12 items were measured in “Yes” or „No‟ responses. Yes was given a value of „1‟ and No was given „0‟. CFA was done to create a composite index (score) and respondents who scored above or equal to the median value were considered to have good neonatal care, while those who scored below the median value were considered as poor neonatal care practice(2).

Knowledge on neonatal care: Twelve questions will be used to measure the knowledge of the respondents on neonatal care. If respondents got the right answer, it was coded as yes "1" if not it was coded as no"0". The knowledge score was developed through Principal Component Analysis (PCA) and the knowledge index was developed and was treated as a continuous variable(8).

Mobile health (mHealth): Mobile health (mHealth) refers to the employment of wireless, moveable data and communication technologies to support health and health care. For this study, mobile health includes sending message service (SMS) on neonatal care for behavior change intervention and reminder(28).

Attitude: Ten questions with a 5 Likert scale will be used to measure the attitude of women to neonatal care. Respondents who scored above or equal to the median value was considered to have a positive attitude and scored below the median value were considered to have a negative attitude to neonatal care practice(29).

Women autonomy: We use 23 items applied considering the three categories namely decision-making autonomy, movement autonomy, and financial autonomy. Those who had median and above values were taken as autonomous (30).

Ethical issues and consideration

This study will adhere to the Helsinki Declaration and will receive ethical approval from the Ethical Review Committee of Jimma University, Institute of Health (Reference Number JUIH/IRB 229/22). Written permissions will be obtained from relevant authorities in Dessie and Kombolcha. After approval, the principal investigator will acquire three SIM cards from Ethio Telecom for the SMS-based intervention. Participants will be informed about the study's aims, their rights, and the importance of their participation, and written informed consent will be obtained. Participation will be voluntary, and participants will be able to withdraw at any time. For those unable to read the messages, arrangements will be made for a trusted family member or husband to read them. If participants do not own a mobile phone but a household member does, they will be linked with the mobile owner. Interviews will be conducted privately, and no personal identifiers will be recorded to maintain confidentiality and anonymity.

Plan for dissemination

The study findings will be deseminated through target Journals and Publications, academic conferences and symposia, develop policy briefs summarizing the key findings, Workshops and Seminars, Collaboration with Professional Networks and Community Outreach.

List of acronyms/Abbreviations

ANC: Antenatal care, CFA:Confirmatory Factor Analysis, EDHS: Ethiopia Demographic and Health Survey, EPPMFP: Early Post-Partum Modern Family Planning, FMOH: Federal Ministry of Health FP: Family Planning, HEP: Health Extension Program, HEW: Health Extension Workers, ID: Identification Number, MPH: Master of Public Health, NNC: Neonatal Care, NNCP: Neonatal Care Practice, ODK: Open Data Kit, PCA: Principal Component Analysis, PI: Principal Investigator,SBA: Skilled Birth Attendant, SE: Standared Error, SEM: Structural Equation Modeling, SDG: Sustainable Development Goals, TBA: Traditional Birth Attendants, WHO: World Health Organization

Declarations

Consent to participants: Informed written consent will be obtained from all subjects before the study.

Consent for publication: Not applicable

Availability of data and material: All data underlying the findings described in the manuscript will be freely available to other researchers within the manuscript itself and uploaded as supplementary information.

Competing interests: The authors declare that they have no competing interests exist.

Funding

The study will be funded by Jimma University, Institute of Health. The funders had no role in study design, data collection and analysis, decision to publish, or preparation of the manuscript.

Acknowledgment

Our sincere gratitude and appreciation go to Jimma University, institute of Health, Department of Population and Family Health with the doctoral program office. Our special thanks go to local authorities,

Authors' profile and contributions

Niguss Cherie (MPH/RH, Assistant Professor), Muluemebet Abera (Professor, PhD), and Gurmesa Tura (Professor, PhD). Conceptualization**:** Niguss Cherie, Muluemebet Abera Wordofa, Gurmesa Tura Debelew. Formal analysis**:** Niguss Cherie.Funding acquisition**:** Muluemebet Abera Wordofa, Gurmesa Tura Debelew. Investigation**:** Niguss Cherie. Methodology**:** Niguss Cherie. Project administration**:** Niguss Cherie, Muluemebet Abera Wordofa, Gurmesa Tura Debelew. Software**:** Niguss Cherie. Supervision: Muluemebet Abera Wordofa, Gurmesa Tura Debelew. Validation: Muluemebet Abera Wordofa, Gurmesa Tura Debelew. Visualization: Muluemebet Abera Wordofa. Writing – original draft: Niguss Cherie. Writing – review & editing: Niguss Cherie, Muluemebet Abera Wordofa, Gurmesa Tura Debelew.

References

1. Report A. For every child, every right. 2018.

2. Tura G, Fantahun M. Neonatal care practice and factors affecting in Southwest Ethiopia : a mixed methods study. 2015;1–10.
3. Ma LH, Alexander M, You D, Alkema L, Group UNI, Estimation M. Articles National , regional , and global levels and trends in neonatal mortality between 1990 and 2017 , with scenario-based projections to 2030 : a systematic analysis. Lancet Glob Heal [Internet]. 2019;7(6):710–20. Available from: http://dx.doi.org/10.1016/S2214-109X(19)30163-9
4. Countries d. the least developed countries report 2020 Productive capacities for the new decade. 2020.
5. Bee M, Shiroor A, Hill Z. Neonatal care practices in sub-Saharan Africa : a systematic review of quantitative and qualitative data. 2018;1–12.
6. Settings L. Target Product Profiles. 2020;(March).
7. Saaka M, Ali F, Vuu F. Prevalence and determinants of essential newborn care practices in the Lawra District of Ghana. 2018;1–12.
8. Memon J, Holakouie-naieni K, Majdzadeh R, Yekaninejad MS. Knowledge , attitude , and practice among mothers about newborn care in Sindh ,. 2019;0:1–9.
9. Report I. Harnessing Frontier Technologies for Sustainable Development. 2018.
10. Spicer N, Agyepong I, Ottersen T, Jahn A, Ooms G. ‘ It ’ s far too complicated ’ : why fragmentation persists in global health. 2020;1–13.
11. Health A. Global investment is needed so that countries can reduce neonatal mortality to below 12 deaths per 1000 live births by 2030. 2021;14–6.
12. Strategic WHO, Framework C, For P, Communications E. Table of Contents.
13. Alamneh Y, Adane F, Yirga T, Desta M. Essential newborn care utilization and associated factors in Ethiopia : a systematic review and meta-analysis. 2020;7:1–9.
14. Mersha A, Assefa N, Teji K, Shibiru S, Darghawth R, Bante A. Essential newborn care practice and its predictors among mother who delivered within the past six months in Chencha District ,. 2018;1–17.
15. Fenta SM, Biresaw HB, Fentaw KD. Risk factor of neonatal mortality in Ethiopia : multilevel analysis of 2016 Demographic and Health Survey. 2021;
16. CSA, demohraphic and health survey Survey H. Ethiopia. 2011;
17. CSA, demohraphic and health survey Survey H. Ethiopia.2016.
18. CSA, Demographic M, Survey H. Ethiopia. 2019.
19. Semanew Y, Etaye M, Tizazu A, Abebaw D, Gebremedhin T. Newborn care practices and its determinants among postnatal mothers in Dessie Referral Hospital , Northeast Ethiopia. BMC Res Notes [Internet]. 2019;1–6. Available from: https://doi.org/10.1186/s13104-019-4133-3
20. Efa BW, Berhanie E, Desta KW, Hinkosa L, Fetensa G, Etafa W, et al. Essential new-born care practices and associated factors among post natal mothers in Nekemte City , Western Ethiopia. 2020;184:1–12.

Available from: http://dx.doi.org/10.1371/journal.pone.0231354

21. Abejirinde IO, Ilozumba O, Marchal B, Zweekhorst M, Dieleman M. Mobile health and the performance of maternal health care workers in low- and middle-income countries : A realist review. 2018;

22. Balakrishnan R, Gopichandran V, Chaturvedi S, Chatterjee R. Continuum of Care Services for Maternal and Child Health using mobile technology – a health system strengthening strategy in low and middle income countries. BMC Med Inform Decis Mak [Internet]. 2016;1–8. Available from: http://dx.doi.org/10.1186/s12911-016-0326-z

23. Nasution LA, Tutik R, Hariyati S. Mobile Health Application in Implementation of Maternity Nursing Care : Literature Review Studi Literatur : tentang Implementasi Aplikasi " Mobile Health " di Pelayanan Keperawatan Maternitas. 1(February 2018).

24. Hackett K, Lafleur C, Nyella P, Ginsburg O, Lou W, Sellen D. Impact of smartphone-assisted prenatal home visits on women ' s use of facility delivery : Results from a cluster-randomized trial in rural Tanzania. 2018;1–20.

25. Modi D, Dholakia N, Id RG, Id SV, Id KD, Id SS, et al. mHealth intervention " ImTeCHO " to improve delivery of maternal , neonatal , and child care services — A cluster-randomized trial in tribal areas of Gujarat , India. 2019;1–24. Available from: http://dx.doi.org/10.1371/journal.pmed.1002939

26. Srikantiah S, Mahapatra T. workers to promote reproductive , maternal , randomized controlled Trial in Bihar , India. 2019;9(2).

27. Shiferaw S, Workneh A, Yirgu R, Dinant G, Spigt M. Designing mHealth for maternity services in primary health facilities in a low-income setting – lessons from a partially successful implementation. 2018;9:1–15.

28. Hall CS, Fottrell E, Wilkinson S, Byass P, Hall CS, Fottrell E, et al. Assessing the impact of mHealth interventions in low- and middle-income countries – what has been shown to work? 2014;9716.

29. Agonafir M, Shimbre MS, Hussen S, Temesgen G, Girmay G, Tsegaye B, et al. Community Based Essential Newborn Care Practices and Associated Factors among Women Who Gave Birth at Home in Last Twelve Months in Amaro Woreda, Southern Ethiopia, 2019. 2019;1–20.

30. Dangal G, Hospital KM, Kutty R. Construction and Validation of a Women ' s Autonomy Measurement Scale with Reference to Utilization of Maternal Health Care Services in Nepal. 2015;(May 2017).

31. Dessie and Kombolcha town adminstration health departments annual plan, 2022

32. Civelek ME. Essentials of structural equation modeling, 2018.

Annex I: Mobile health intervention schedule and package (Protocol)

Table10**:** Intervention schedule and package/Protocol on effectiveness of mobile health intervention to improve neonatal care practice at Dessie and Kombolcha, north east Ethiopia.

**Sr. No**	**Week**	***Intervention package/Protocol***
1	30th weeks of gestation	*"Good health to you. "Dear sir, mother do the following accordingly to improve the health of the mother and neonate, Follow at least 4 ANC visits at health facilities, Take TT vaccination, Take Iron foliate, plan to give your birth at health facilities, Plan to take birth control method to take early after child birth to prevent short birth interval. If you have any question/need of clarification related the message you can miscall/call with this mobile number.*
2	32th weeks of gestation	*"Good health to you. "Dear sir, mother do the following accordingly to improve the health of the mother and neonate, Follow at least 4 ANC visits at health facilities, Take TT vaccination, Take Iron foliate, plan to give your birth at health facilities, Plan to take birth control method to take early after child birth within 45 days after delivery to prevent short birth interval. If you have any question/need of clarification related the message you can miscall/call with this mobile number.*
3	34th weeks of gestation	*"Hello good health to you. "Dear Sir, there is a need of birth spacing minimum of 2-3 years. Sometimes a woman can be pregnant starting from 45 days after child birth even without showing menstruation if she has sexual practice. To prevent this decide to take early postpartum family planning after child birth any time starting from immediately after birth to 45 days after". If you have any question/need of clarification related the message you can miscall/call with this mobile number.*
4	36th weeks of gestation	*"Hello good afternoon. The time to delivery is reaching and does the following. Decide to give birth at health facility, Prepare emergency transport, and Prepare social support to health facility. After delivery make skin to skin contact with the new born, Exclusive beast feed with in 1hr after birth, Do not bath the new born with in 24 hrs, Feed colostrum to the new born, Do not apply anything on the cord, Do post natal check up, follow immunization of the child on the date of birth and after that". If you have any question/need of clarification related the message you can miscall/call with this mobile number.*
5	38th weeks of gestation	*"Good health to you. The time to delivery is reaching and does the following. Decide to give birth at health facility, Prepare emergency transport, and Prepare social support to health facility. After delivery make skin to skin contact with the new born, Exclusive beast feed with in 1hr after birth, Do not bath the new born with in 24 hrs, Feed colostrum to the new born, Do not apply anything on the cord, Do post natal check up, follow immunization of the child on the date of birth and after that". If you have any question/need of clarification related the message you can miscall/call with this mobile number.*
6	40th weeks of gestation	*Dear sir mother, the following are key activities to keep the health of your neonate. Make skin to skin contact with the new born after birth, Exclusive beast feed with in 1hr after birth, Do not bath the new born with in 24 hrs, Feed colostrum to the new born, Do not apply anything on the cord, do post natal check up to check the status of the child, follow immunization of the child on the date of birth and after that". To prevent narrow birth interval and unwanted pregnancy decide to take early postpartum family planning after any time starting from immediately after birth to 45 day". If you have any question/need of clarification related the message you can miscall/call with this mobile number.*
7	2nd week postpartum	*Dear sir mother, the following are key activities to keep the health of your neonate. Make skin to skin contact with the new born after birth, Exclusive beast feed with in 1hr after birth, Do not bath the new born with in 24 hrs, Feed colostrum to the new born, Do not apply anything on the cord, do post natal check up to check the status of the child, follow immunization of the child on the date of birth and after that". To prevent narrow birth interval and unwanted pregnancy discuss among your husband and decide to take early postpartum family planning after any time starting from immediately after birth to 45 day". If you have any question/need of clarification related the message you can miscall/call with this mobile number.*
8	4th week postpartum	*Dear sir mother, the following are key activities to keep the health of your neonate. Make skin to skin contact with the new born after birth, Exclusive beast feed with in 1hr after birth, Do not bath the new born with in 24 hrs, Feed colostrum to the new born, Do not apply anything on the cord, do post natal check up to check the status of the child, follow immunization of the child on the date of birth and after that". To prevent narrow birth interval and unwanted pregnancy discuss among your husband and decide to take early postpartum family planning after any time starting from immediately after birth to 45 day". If you have any question/need of clarification related the message you can miscall/call with this mobile number.*
